# Advancing the assessment of clinical reasoning across the health professions: Definitional and methodologic recommendations

**DOI:** 10.1007/s40037-022-00701-3

**Published:** 2022-03-07

**Authors:** David Gordon, Joseph J. Rencic, Valerie J. Lang, Aliki Thomas, Meredith Young, Steven J. Durning

**Affiliations:** 1 Division of Emergency Medicine, Duke University, Durham, NC USA; 2 Department of Medicine, Boston University School of Medicine, Boston, MA USA; 3 Division of Hospital Medicine, University of Rochester School of Medicine and Dentistry, Rochester, NY USA; 4 School of Physical and Occupational Therapy, Institute of Health Sciences Education, McGill University, Montreal, QC Canada; 5 Department of Medicine and Institute of Health Sciences Education, McGill University, Montreal, QC Canada; 6 Department of Medicine, Uniformed Services University of the Health Sciences, Bethesda, MD USA

**Keywords:** Clinical reasoning, Assessment, Health professions, Conceptual frameworks, Validity

## Abstract

The importance of clinical reasoning in patient care is well-recognized across all health professions. Validity evidence supporting high quality clinical reasoning assessment is essential to ensure health professional schools are graduating learners competent in this domain. However, through the course of a large scoping review, we encountered inconsistent terminology for clinical reasoning and inconsistent reporting of methodology, reflecting a somewhat fractured body of literature on clinical reasoning assessment. These inconsistencies impeded our ability to synthesize across studies and appropriately compare assessment tools. More specifically, we encountered: 1) a wide array of clinical reasoning-like terms that were rarely defined or informed by a conceptual framework, 2) limited details of assessment methodology, and 3) inconsistent reporting of the steps taken to establish validity evidence for clinical reasoning assessments. Consolidating our experience in conducting this review, we provide recommendations on key definitional and methodologic elements to better support the development, description, study, and reporting of clinical reasoning assessments.

## Background

Clinical reasoning broadly entails the cognitive processes that enable clinicians to observe, collect, and analyze information, resulting in decisions and actions that seek to improve patients’ well-being while considering their specific circumstances and preferences [1, 2]. Clinical reasoning is a multidimensional construct that is inherently complex [2], comprised of different components (e.g., data gathering, problem representation) [3], and undergirded by varied theoretical frameworks and epistemologies [1]. More recently, the conceptualization of diagnosis as a team-based activity has emerged, emphasizing clinical reasoning as a collaborative effort between health care professionals, patients, and families [4, 5].

The health professions encompass a broad array of practices which share a common commitment to the study, diagnosis, treatment, and prevention of human illness and injury [6]. In fulfilling this purpose, the health professions have recognized the centrality of clinical reasoning and its assessment, as represented in multiple competency-based educational frameworks [7–10]. From the patient safety perspective, faulty reasoning has been identified as a major cause of diagnostic error [11], and thus strategies to improve clinical reasoning education and assessment and reduce clinical reasoning errors have been highlighted as an important area for health professions education (HPE) and research [12, 13].

To understand how clinical reasoning is assessed across the health professions, our group of clinical educators and researchers undertook a scoping review of the HPE literature [14], which in turn led to additional work on how clinical reasoning is variably defined and conceptualized [15, 16]. In the process of synthesizing the vast body of literature across multiple health professions we encountered multiple challenges which prompted us to reflect on our experience and identify opportunities for better definitional and methodologic clarity in clinical reasoning assessment research and reporting. Clinical reasoning is a topic that often feels familiar (or even obvious) as it permeates health professions education, however, as we observed, this sense of familiarity may be masking important differences in how it is understood, operationalized, and assessed.

The overarching aim of this eye opener is to share the challenges we had in synthesizing a large, and somewhat fractured, body of literature on a multidimensional construct and then propose recommendations to better support the development, description, study, and reporting of assessments. The recommendations that we include in this eye-opener are generated based on our collective experience but draw from and align with sound research and assessment practices in terms of the importance of conceptual frameworks [1, 17–19], detailed assessment methodology [20, 21], and sources of validity evidence [22–24] in modern educational assessments. While our experiences were deeply embedded in the clinical reasoning context, we believe that similar complexities are likely encountered when synthesizing other multidimensional constructs in health professions education, and as such, our recommendations may be of value in other literature spaces.

Following a description of the challenges we experienced synthesizing literature in clinical reasoning assessments, we present resultant experienced-based and best-practices-linked recommendations. Through this synthesis, we seek to bring new perspective and collective insight into the current state and future direction of clinical reasoning assessment by first illustrating the need for greater clarity in the HPE literature followed by proposing a roadmap for how this can be achieved.

## Observations from a scoping review of clinical reasoning assessment

Our author team conducted a large scoping review on clinical reasoning assessment. A scoping review is a methodology well suited to mapping literature that may be evolving, disperse, or contain a variety of different conceptualizations [25–27]. We used a mapping approach for our synthesis given the diversity of clinical reasoning assessment approaches—from knowledge-based exams to workplace-based assessments. While we expected the literature on clinical reasoning assessment to be varied, we found its synthesis particularly challenging because: 1) clinical reasoning is a multidimensional construct with varied conceptualizations and definitions, 2) method sections often lacked adequate detail, and 3) there was a paucity of validity evidence supporting a particular clinical reasoning assessment approach.

Several observations regarding the conceptualization of clinical reasoning have emerged [14]. The presence of multiple conceptualizations can result in different boundaries as to what is considered clinical reasoning, different terms being used that refer to the broad construct of clinical reasoning, and the same term being used with different intended interpretations. First, different understandings of what “counts” as relevant to clinical reasoning exist, which can result in different definitions, and influences what might be a relevant target for assessment [15]. For example, conceptualizations that focus on the cognitive processes within individuals can lead to one definition with one set of targets for assessment, while those that emphasize the interplay between provider, patient, and environment can result in another definition and create a different set of targets. Second, numerous terms have emerged in the literature that relate to clinical reasoning. One study identified 110 different terms used in reference to the construct of clinical reasoning [16]. Some of these terms, such as “clinical skills” and “competence,” are very broad and, without an accompanying clear definition, require inferences on the part of a reader to determine the intended assessment focus. More specifically, these broad terms make it challenging to determine how clinical reasoning has been operationalized into a target for assessment, or whether an ability traditionally regarded as a different domain—like procedural or communication skills [7, 8, 15]—is the focus of the assessment. Lastly, the same term can take on different meanings. For instance, in the field of surgery, “judgment” has been used to represent “respect for tissue” and assessed by measuring the amount of force applied [28]. In other medical fields, judgment has been used to represent sound problem solving and decision making within a given clinical context [29]. In further contrast, in the field of nursing, the emphasis has been placed on judgment as the *outcome* of a reasoning process [10]. Therefore, the same term has been used to refer to a specific subskill, a transferable set of approaches, and an outcome of the reasoning process. This multiplicity of meanings across contexts, especially when not accompanied by an explicit definition, makes the mechanisms of synthesis challenging, and more importantly relies heavily on a reader being aware of, and responding to, different contextually-bound uses of the same term.

Our scoping review also provided insight into the methodological and reporting rigor in literature describing clinical reasoning assessments. One key means of documenting the potential value of an assessment tool is attending to, collecting, and reporting on validity evidence. Despite its importance, few papers in our review used any explicit validity framework or provided validity evidence to support the use of their assessment. The infrequency of validity evidence included in an assessment paper in the HPE literature is not unique to clinical reasoning. Others have reported similar findings of limited validity evidence in technology-enhanced simulation-based assessments—primarily focused on procedural skills but also used to evaluate communication and teamwork skills [30]—and in professionalism assessments [31].

Many key concepts in HPE are complex and often multidimensional. Clinical reasoning is not unique in its complexity but provided us with some deeply grounded experience in synthesizing the literature on an important but often inconsistently operationalized focus of assessment in HPE. Considering our experience, we would like to suggest a series of experience-based and best-practices-linked recommendations focused on the use of more explicit terminology, incorporation of theoretical framework(s), increased methodologic detail, and the provision of validity evidence. We believe that these recommendations developed in the context of clinical reasoning assessment may provide helpful guidance for others who study, teach, or assess other broad concepts (e.g., professionalism, expertise) in HPE.

## Recommendations

### Define the construct of interest

Clearly defining a construct is essential to assessment development and validation [20, 23]. When considering a broad concept like clinical reasoning, it is important that authors provide an explicit definition of clinical reasoning (or synonym) or a clear description of the target construct used in their assessment. This recommendation does not imply that there is one universally “correct” definition of clinical reasoning [32, 33], but rather it emphasizes the importance of providing a clear description of the specific construct of interest. This clear description will promote appropriate understanding, defensible application, and meaningful integration of the assessment method.

In developing a construct of clinical reasoning for assessment, an important consideration to specify is whether clinical reasoning is being assessed as an outcome, process, or combination thereof [15]. This clarification of clinical reasoning as an outcome, process, or both leads to the use of different forms of assessment. For example, an assessment concerned with diagnostic accuracy may treat clinical reasoning as an outcome (i.e., a defined output) while another assessment concerned with data gathering may emphasize clinical reasoning as a process (i.e., a sequence of steps). How well the assessment distinguishes between process and outcome, and how that relates to the construct definition, will have important implications in understanding the strengths and limitations of a given assessment approach.

If an assessment is a process-oriented assessment (i.e., focused on clinical reasoning as a process rather than an outcome), authors should clearly define the clinical reasoning subprocesses of interest [3, 14]. Some assessments focus on information gathering and differential diagnosis (e.g., OSCEs); other methods are geared towards the assessment of problem representation (e.g., concept maps) [14]. Specifying the clinical reasoning subprocesses or components targeted by a given assessment approach will make it clear *if, how, *and *in which manner *the assessment method is capturing clinical reasoning performance.

Clinical reasoning has been studied using a variety of lenses, and several theories have been mobilized to better understand this complex construct. Given that clinical reasoning can be considered a process or outcome, a deeply cognitive or collaborative activity, we recommend that authors describe the theoretical framework(s) that shape how they understand the construct of clinical reasoning, and the perspectives that ground the assessment and study design [34]. Multiple theoretical frameworks exist that highlight the multiple understandings of clinical reasoning, and different frameworks provide different rationales for selecting certain assessment approaches over others [19]. For example, use of dual-process theory as a conceptual framework may lead researchers to discriminate between and assess both unconscious/automated cognitive processes and conscious/analytical processes [35]. Alternatively, relying on situated cognition theories may lead to emphasizing a contextual dimension to clinical reasoning—as a process that emerges from interactions between physician factors, patient factors, and practice environment factors [36]. If these two different theoretical stances are carried forward to assessment design, one could imagine a situated cognition researcher focusing an assessment approach on how the diagnostic accuracy of a learner is affected by an angry patient, whereas a researcher using dual process theory might focus on the cognitive processes a learner uses to navigate diagnostic ambiguity. Explicitly stating the theoretical framework and definition of clinical reasoning in an article allows the reader to understand the rationale for the assessment methodology and discussion of the results.

### Describe the assessment tool

In addition to an explicit definition or articulated construct, a clearly described assessment development and validation approach is critical to understanding the nature, purpose, quality, and potential utility of the assessment. Clinical reasoning assessments can vary by their setting (classroom versus clinical), type of encounter (simulated versus workplace), clinical reasoning component of interest, and a variety of other factors [14, 37, 38]. Additional important components of an assessment include the instruments used to collect examinee responses (i.e., assessment format), the method used to score those responses, the intended use of the scores generated by the assessment, and, when applicable, the background or training of the raters who conduct the scoring.

In conducting our review on assessments of clinical reasoning, our research group focused on data reported about an assessment that included: the stimulus format (i.e., what necessitated a response), response format (i.e., how a learner could respond to the assessment), and scoring activity (i.e., how a learner response was transformed into an assessment score). We found these elements helpful in structuring our analysis of existing literature [14] and suggest reporting these characteristics would increase clarity for the development and description of new research on clinical reasoning assessment.

*Stimulus format *describes the way in which an examinee is presented with a clinical scenario [21]. Examples include a real patient, standardized patient, computer-based virtual patient, or a written clinical vignette. Providing detail on how these stimuli are chosen or constructed, their level of complexity, and their degree of intended ambiguity or uncertainty will help promote understanding of the development, scope, and application of the assessment and contribute to building a validity argument in support of score interpretation [38].

An examinee’s choices or series of actions in response to the stimulus format need to be recorded, and this component is captured in *response format *[21]. Responses can be *selected* in which an examinee chooses from a list of provided answers or *constructed* in which an examinee responds verbally or in writing to open prompts [21]. Furthermore, constructed responses can exist in different formats such as essays, diagrams, and post-encounter documentation. We recommend authors describe how response instruments were either created or selected to meet the goals of the assessment and—particularly if novel—whether they were piloted to see if examinees understood the question, response options, and mechanics of the collection instrument.

*Scoring activity* is the process by which examinee’s responses are converted into a performance result. It can be quantitative or qualitative. It refers to both “answer key” generation and application, and in certain assessment approaches includes rater training. Scoring activities can occur pre-, intra-, and/or post-assessment and should be explicitly described. Answer key generation typically occurs pre-assessment, when a group develops a clinical question or scenario and determines the “correct” response. Information on how consensus was achieved for the answer key (if relevant) should be provided.

Intra-assessment scoring occurs primarily during direct observation. Authors should provide the details about the tool with accompanying rationale for why a particular scoring approach is used (e.g., checklist vs global; complete/incomplete vs. Likert-scale). Intra-assessment scoring activity is challenging because of the multi-tasking required of the assessor. Thus, authors should provide details about rater qualifications, experience, training, and inter- or intra-rater reliability. Information should also be provided about the time needed to complete the assessment activity.

Post-assessment scoring can involve grading selected or constructed responses. Depending on the format, it may be automated or by hand. Like intra-assessment scoring, providing background information on the scorers and/or technology and time needed to complete the activity will allow for determinations about the feasibility of an assessment method.

In summary, to better support transparent reporting, we suggest papers describing assessments of clinical reasoning include a clear definition of the construct of interest, describe the theoretical framework underpinning the assessment, and describe the stimulus format, response format, and scoring activity.

### Collect validity evidence

Validity refers to the evidence provided to support the interpretation of the assessment results and the decisions that follow [23]. Evidence of validity is essential to ensure the defensible use of assessment scores and is an important component of assessment literature [39]. A common misassumption that we encountered in our scoping review was that validity is a dichotomous feature (i.e., that an assessment is either valid or not valid). This notion of validity as a characteristic of a test has been reported elsewhere and is not limited to the clinical reasoning assessment literature [40]. In more current conceptualizations of validity, an assessment may have varying strengths of evidence in an argument supporting the use of its scores to make specific inferences about specific populations. In other words, evidence of validity does not “travel” with an assessment tool and should be collected each time an assessment is used in a different context or population and for each different score use (i.e., formative feedback vs. summative decisions). For example, validity evidence collected for an examination to determine whether medical students demonstrate a minimum level of competence in their clinical reasoning to pass the pediatrics clerkship would not be sufficient to justify using the examination to determine whether pediatric residents should be licensed to practice independently. In summary, validity evidence must be collected for a clinical reasoning assessment supporting the intended score use and the decisions that result.

We recommend that authors use an explicit validity framework to collect and report the validity evidence. There are multiple approaches, but in HPE, two major validity frameworks are frequently used: Messick’s unified framework of construct validity [41] and Kane’s validity argument framework [42]. Messick’s framework, as modified by Downing [23], identifies five categories of evidence to support score interpretation and use. These categories (adapted in the *Standards for educational and psychological testing* [39]) are: content (e.g., blueprinting an examination or ensuring workplace assessments are performed aligned with the definition of clinical reasoning adopted), response process (e.g., ensuring those being assessed and those assessing understand the assessment as intended), internal structure (e.g., analysis of difficulty and discrimination of items/cases and how that aligns with the definition of clinical reasoning adopted), relationship to other variables (e.g., relationship with another assessment that assesses a similar or different aspect of clinical reasoning), and consequences (e.g., whether decisions made based on results of the assessment are justified for the individual and the institution). Notably, reliability is included as one piece of evidence supporting validity (part of internal structure), rather than treated as a separate construct from validity. In summary, Messick’s approach focuses on the types of evidence that help us determine whether a decision based on assessment data is sound, or, in other words, whether a score interpretation (e.g., pass/fail) is defensible.

As described by Schuwirth and van der Vleuten, Kane’s framework involves organizing categories of evidence into a logical argument to justify that the units of assessment can be translated into an inference about the population being assessed [24]. The four links in the logic chain are scoring (translation of an observation into a score), generalization (using a score to reflect performance in a particular setting), extrapolation (using the score to reflect performance in the real-world setting), and implications (using the score to make a decision about the learner). Through this lens, sequential inferences are made about the learner’s clinical reasoning. For example, a workplace-based assessment may begin with a chart-stimulated recall [43] of a resident’s clinical reasoning about a case (a supervisor translates that observation into a score), followed by generalization of that score to make an inference about the resident’s clinical reasoning performance in the outpatient clinic within their patient population, followed by extrapolation to their clinical reasoning ability for outpatients in general, and ending in a decision to allow the resident to make clinical decisions without supervision.

Our recommendations and resources for developing, describing, and documenting an assessment of clinical reasoning are summarized in Tab. 1. Where Messick’s approach focuses on types of evidence, Kane focuses on the types of inference that are made moving from an assessment to judgment such as “competent.” Thus, these two frameworks are complementary and have been combined in order to think comprehensively about the validity of assessment score interpretation [44].

**Table 1:** Summary of recommendations for developing and detailing an assessment of clinical reasoning

Recommendation	Example	Resources
**Definition/Conceptualization** Provide an explicit definition or conceptualization of clinical reasoning being used in the assessment	“For the purpose of this paper, clinical reasoning is defined as a *skill, process*, or *outcome* wherein clinicians observe, collect, and interpret data to diagnose and treat patients” [14, p. 902]	[2, 32, 33]
**Theories of clinical reasoning/Conceptual frameworks** Incorporate and discuss any theoretical frameworks that inform the conceptualization of clinical reasoning and its assessment	Script theory is provided as the theoretical basis for the script concordance test [45]	[1, 17–19]
**Assessment methodology** Provide specific descriptions of the stimulus format, response format, scoring activity, and rater information	Methodology includes detailed description of standardized patient exam with free text response, evaluated by physician raters using a grading protocol [46]	[20, 21]
**Validity** Describe sources of validity evidence using an established framework, including reliability measures	Content, response process, and internal structure provided as sources of validity evidence for an instrument evaluating virtual patient design [47]	[22–24]

We hope these recommendations can assist investigators in their conception and execution of new studies on the assessment of clinical reasoning. Similarly, these recommendations can guide educators in analyzing the quality of existing studies in order to decide whether to incorporate an assessment approach into their own educational program. Towards this end, these recommendations offer a structured approach to question the utility of an assessment paper based on whether a clear definition of clinical reasoning has been provided in order to evaluate if the study is generalizable to one’s context, a theory was utilized to shed insight into the perspectives and assumptions behind the study, sufficient methodological detail is available for the assessment to be reproduced accurately and reliably, and the validity evidence provides adequate justification for pass/fail or promotion decisions.

Potential future implications of our reflections and proposed recommendations include the creation of formal guidelines to be used in reporting of studies on the assessment of clinical reasoning, similar to PRISMA guidelines for systematic reviews and metanalysis [48]. Our recommendations require further review and discussion by experts in clinical reasoning and assessment research as well as additional empirical examination, but we hope this manuscript can serve as an important conversation starter for developing formal guidelines. As the volume of articles on clinical reasoning assessment continues to rapidly expand [16], we worry that the literature will become even more fragmented, further impeding the ability to synthesize, reproduce, and generalize research in this critical domain. An increasingly fragmented literature may limit our ability to effectively share innovative approaches to assessment, and limit constructive engagement with how to best assess, or how to best combine multiple assessments, to effectively capture clinical reasoning performance.

## The future of clinical reasoning

As health care delivery continues to evolve, so likely will the dimensions of clinical reasoning. The emergence of health systems science as a third pillar in medical education will place increased emphasis on systems thinking and caring for both populations and individuals [49]. With the growth of electronic health records and big data, computational thinking and data literacy can be expected to be of increased importance in clinical decision making [50]. Telehealth has provided a new environment in which clinical reasoning can be situated [51]. These expanded possibilities of what clinical reasoning entails and the contexts in which it can occur will require ongoing discussion on how different conceptualizations of clinical reasoning can be captured and assessed. This evolution of clinical reasoning further stresses the importance for definitional and methodologic clarity in future studies on clinical reasoning assessment to facilitate synthesis across research and promote cohesion in the HPE literature.

## Conclusion

To better support researchers, educators, and assessors, we suggest a variety of means to increase the quality of reporting on assessments of clinical reasoning. Including an explicit definition, a description of the theoretical frameworks being applied, and organizing validity evidence within a recognized framework will better support communication about assessments of clinical reasoning and hopefully support more consistent implementation and use of novel approaches to assessment. Our recommendations represent the application of several general principles of educational assessments to the design, documentation, and reporting of clinical reasoning assessments. They can easily be applied to other domains of study in HPE, particularly those involving constructs that are broad in their scope, open to different interpretations, and comprised of multiple components.
